# “More air—better performance—faster recovery”: study protocol for randomised controlled trial of the effect of post-stroke inspiratory muscle training for adults

**DOI:** 10.1186/s13063-021-05551-8

**Published:** 2021-08-28

**Authors:** Susanne Lillelund Sørensen, Simon Svanborg Kjeldsen, Sine Secher Mortensen, Ulla Torp Hansen, Dorthe Hansen, Asger Roger Pedersen, Hanne Pallesen

**Affiliations:** Hammel Neurorehabilitation Centre and University Research Clinic, RM, University of Aarhus, Voldbyvej 15, 8450 Hammel, Denmark

**Keywords:** Inspiratory muscle training, Fatigue, Stroke, Endurance in gait, Voice volume and phonation, Activity of daily living, Spirometry, Peak expiratory flow

## Abstract

**Background:**

Stroke results in varying physical, cognitive, emotional and/or social disabilities in the short and long term alike. Motor impairments are important, persistent consequences of stroke and include, among others, decreased respiratory muscle function, decreased ability to expand the thorax and postural dysfunction. These deficits affect the patient’s ability to perform daily activities, produce fatigue and reduce endurance and quality of life. Inspiratory muscle training (IMT) aims to improve the strength and endurance of the diaphragm and the external intercostal muscles. The objectives of this study are to investigate the effect of 3 weeks of IMT on (i) maximal inspiratory pressure (MIP) in adults having suffered a stroke, as well as (ii) functional activities and expiratory measurements.

**Methods/design:**

This is a randomised controlled trial (RCT) comparing IMT with conventional neurorehabilitation (usual practice). The trial will include 80 patients with reduced MIP hospitalised at a specialised neurorehabilitation hospital in Denmark. The intervention group will receive IMT sessions, exercising at 30% of MIP. Patients in the intervention group will perform two daily sessions (one session of IMT with Threshold IMT consisting of two times 15 inspirations at normal breathing rhythm (5–10 min)), 7 days a week for 3 weeks. Training can be with or without physiotherapist supervision. Study outcomes: MIP assessed by the Power Breath, Functional Independence Measurement, The 6-min walk test, the Fatigue Severity Scale and average voice volume. Expiratory function will be assessed using spirometry. All assessments will be conducted at baseline and 3 weeks (at termination of the intervention) and 3 months after the intervention has concluded.

**Discussion:**

IMT is a promising and partly self-managed tool for rehabilitation to improve respiratory function. The introduction of IMT in combination with traditional physical therapy may enhance faster recovery after stroke and may at the same time demand little personnel resources to increase training intensity. This trial will provide further evidence of IMT to clinicians, patients and health managers. Hereby, this study accepts the call for further research.

**Trial registration:**

ClinicalTrials.govNCT04686019. Registered on 28 December 2020.

**Supplementary Information:**

The online version contains supplementary material available at 10.1186/s13063-021-05551-8.

## Background

In Denmark (counting approximately 5.8 million inhabitants [1]), 12,500 people experience stroke annually [2]. The incidence is highest in the elder part of the population. As the average life expectancy increases, stroke is expected to become an even heavier burden on the healthcare system [2, 3]. Stroke results in varying physical, cognitive, emotional and/or social disabilities in the short and long term alike [4, 5]. Motor impairments are significant and persistent consequences of stroke and include decreased respiratory muscle function, decreased ability to expand the thorax and postural dysfunction, among others [6–9]. These deficits affect the patient’s ability to engage in daily activities, produce fatigue, reduce endurance and affect quality of life.

Inspiratory muscle training (IMT) aims to improve the strength and endurance of the diaphragm and external intercostal muscles [9]. In sports, IMT serves to improve athletes’ respiratory capacity [10, 11]. Smaller studies have tested the method in post-stroke patients, and results indicate that IMT has a positive effect on respiratory muscle function, exercise capacity and quality of life [12–14]. A review recommends using IMT as an intervention for patients suffering from chronic obstructive pulmonary disease (COPD) [15]. In Denmark, IMT has been applied in practice, for instance in persons with tetraplegia in the acute phase. IMT has also been used in treatment of people with COVID-19; however, knowledge of the use of IMT is surprisingly sparse. This project will be one of the first to bridge this knowledge gap. Clinical experiences in neurorehabilitation imply that IMT may improve voice volume, phonation endurance and coughing force (airway clearance). It would be useful to substantiate evidence presented for this hypothesis. It is therefore relevant to test the intervention in a larger study setup.

The aim of this trial is to determine the efficacy of a three weeks IMT intervention in adults post-stroke.

### Objectives

The two primary objectives of this study are to investigate:

1. The effect of 3 weeks of IMT on maximal inspiratory pressure (MIP) in adults having suffered stroke.
2. The effects of 3 weeks of IMT on the degree of dependency in activities of daily living, endurance in gait, fatigue, voice volume (VV), phonation endurance (PE) and expiratory function; peak expiratory flow (PEF), forced expiratory volume in 1 second (FEV1), forced vital capacity (FVC) and FEV1/FVC ratio.

## Methods/design

### Design

This study is a randomised controlled trial (RCT) comparing IMT with conventional neurorehabilitation (usual practice). Baseline assessments are made 0–4 days before the intervention starts. Further assessments are performed 0–4 days after the intervention concludes and at the 3-month follow-up. Assessments will be conducted by assessors blinded to the randomisation and the intervention.

### Patient population

We plan to enrol 80 patients in the study. All patients will be recruited from a specialised neurorehabilitation hospital in Denmark.

### Inclusion criteria

- First-time brain infarction or brain haemorrhage within the past 0–3 months
- Age > 17 years
- Able to give written consent and being cognitively and communicatively able to understand and participate in the MIP test
- Reduced MIP below the gender- and age-specific normal standard given in Table 1. Patients > 74 years of age will be categorised as having reduced MIP according to the subgroup age 70–74 standard values.

**Table 1:** MIP—gender- and age-specific standard

Standard values for maximal inspiratory pressure (MIP)					
Age	20–54	55–59	60–64	65–69	70–74
Men	124 ± 44	103 ± 32	103 ± 32	103 ± 32	103 ± 32
Women	87 ± 32	77 ± 26	73 ± 26	70 ± 26	65 ± 26

### Exclusion criteria

**Fig. 1:**
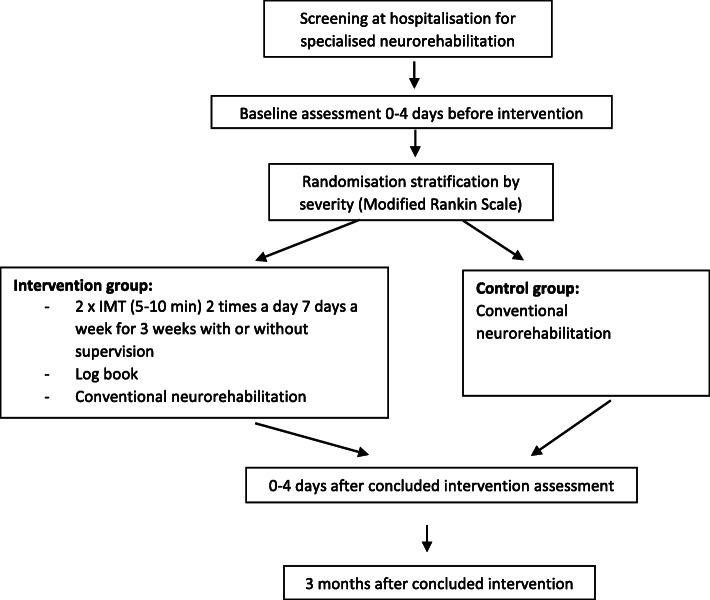
Patient flowchart

Patients with the following characteristics will be excluded: diagnosis of myocardial infarction within the past 3months, significant pulmonary disease (severe COPD), saturation below 90 if having COPD and for others a saturation below 92, neurological deficits other than stroke, facial palsy affecting proper labial occlusion and dizziness or nausea/vomiting during MIP testing (Fig. 1).

### Randomisation

An independently controlled, computer generated randomisation will be implemented in REDCap [16, 17] by an externally based datamanager. The allocation will be concealed to the involved assessors. Only the project management has access to the randomisation information in REDCap and only after each patient has been randomised. The project manager informs the physiotherapist at the ward about the allocation. Afterwards, the physiotherapist informs the patient. A stratified block randomisation of severity of impairment based on Modified Rankin Scale (mRS) (< 4) and a block size of 4 with an intervention/control ratio of 1/3 will be performed. Furthermore, a stratification on the two participating neurorehabilitation units will be performed.

### Intervention

Participants in both groups will receive conventional neurorehabilitation–treatment as usual. This comprises of 45 min physiotherapy sessions 5–7 times per week individual or in groups. Treatment’s focus depends on individually set goals agreed upon in the rehabilitation plan. The intervention group will receive add-on IMT sessions with exercises at 30% of MIP. MIP is measured by instructing the patient to perform five forceful inspirations against an occluded mouthpiece Power Breathe KH2 (POWERbreathe International Ltd, UK). The best score is multiplied by 0.3 to determine resistance. IMT is performed with the Threshold IMT (Philips, UK) with the calculated resistance. During the Power Breath measurement, the patient sits straight up and uses a nose clip. The patient may receive help to hold the Power Breath if necessary.

One session of IMT with the Threshold IMT consists of two times 15 inspirations at normal breathing rhythm (5–10 min). Patients in the intervention group perform two sessions a day (morning and evening); training is performed prior to a meal, 7 days a week for 3 weeks. According to the patients’ need of support, IMT can be with or without supervision. MIP is reassessed every 7 days, and a new resistance is calculated. Patients in the intervention group log their IMT in a log book. The log book supports the patient in performing the right number of respirations twice a day for 3 weeks. The patient sits straight up in a chair/wheelchair/armchair, possibly with a table in front of him or her during practice. The Threshold IMT mouthpiece is placed after the resistance has been set manually. The nose clip must be used during practice to ensure that air passes through the mouth only. Any adverse events are noted in the log book and the staff will contact the project manager. The project manager will in collaboration with the patient and the physiotherapist decide what action to take. As inpatients, the participants in this project are covered by patients’ right to apply for compensation if any unexpected events related to IMT should occur.

### Outcomes

The primary outcome measure is MIP assessed by Power Breath [18]. This is an objective measurement used to describe the inspiratory capacity.

Secondary outcome measures include the following: the Functional Independence Measure (FIM) to assess the degree of dependency in daily activities—both total score and motor sub score [19]—and the 6-min walking test to measure endurance [20–22]. Fatigue was measured by the Fatigue Severity Scale [23, 24]. Average voice volume will be measured by recording of an a-sound with the app Voice Analyst. Phonation endurance (how long the a-sound lasts) is noted. The patient tries three times, and the average is then calculated. Expiratory function is as follows: peak expiratory flow (PEF), forced expiratory volume in 1 s (FEV1), forced vital capacity (FVC) and FEV1/FVC ratio will be assessed with Spirometry device (Vitalograph Micro 6300, Ireland). All respiratory measures are performed in sitting position. The patient tries three times. Subsequently, the best score is used for further analysis [12].

### Test procedure

All assessments will be conducted at baseline, 3 weeks (at termination of the intervention) and 3 months after the intervention concluded. The Patient Global Impression of Change (PGIC) scale will be employed upon conclusion of the intervention [25].

Outcome assessors are blinded to the intervention. Testing takes place in a specific room at the recruitment hospital. Outcome assessors are physiotherapists trained in performing the test procedures and are not joining the treatment team at the recruiting ward. Patients will be tested by the same therapist all three times. The trial participants and intervention providers are not blinded.

### Sample size

Power was calculated to establish the needed number of participants. The power calculation was based partly on a local pre-study (including 26 patients) and on a RCT study [12], which showed differences in variance in ∆MIP assessments. ∆MIP is defined as (MIP at 3 months minus MIP at baseline). Because of the difference in variances in the intervention group and the control group, the same power can be achieved by distributing them unequally between the groups. It is assumed that the standard deviation is three times larger in the intervention group than in the control group. The power calculation prescribes 20 patients in the control group and 60 patients in the intervention group. Calculations were made, expecting a 10% dropout. The least clinically relevant difference was estimated to 5 cmH_2_O.

### Statistical analysis

Data will be analysed as intention-to-treat and per protocol. Between-group differences (3-month follow-up minus baseline) are compared by the *T* test. Regression analysis is conducted with adjustment for the confounders’ age and time from stroke to baseline assessment.

### Data monitoring

The trial will be monitored by the project management, who regularly will follow-up on missing data and progress in the data collection. A formal data monitoring committee is not need in this trial due to its relative small sample size (*n* = 80) and no known harms.

There are no plans for independent auditing the trial, but the investigators will follow-up on missing data in REDCap. Furthermore, the procedures in the protocol will continuously be discussed and evaluated with both the assessors and the physiotherapists implementing the IMT.

### Study organisation

This study is organised and coordinated by the research unit at the Hammel Neurorehabilitation Centre. The participating stakeholders in this study are clinicians and researchers at the Hammel Neurorehabilitation Centre. All parties participate in the Steering Committee and are involved in discussing interim results and making the final decisions about the trial.

## Discussion

The primary purpose of this study is to assess the effect of an IMT intervention in patients with reduced MIP following stroke. A secondary purpose is to assess the effect of IMT on the degree of dependency in activities of daily living, endurance in gait, fatigue, voice volume, phonation endurance and expiratory capacity. Hereby, this study accepts the call for further research made by Xiao [9].

The study assumes that by strengthening the diaphragm muscle and the intercostal muscles, patients will gain faster respiratory recovery, which, in turn, will allow them to practice or carry out activities of daily living earlier in the course of their rehabilitation. The typical length of IMT interventions is 6–8 weeks [12, 13, 26]. In 2019, the average hospitalisation in the study setting for stroke patients was 34 days (95% CI 21–52 days). The length of the intervention was set to 21 days to ensure that the intervention could be completed during hospitalisation. The duration of the intervention was also informed by positive results from the pre-study. We argue that it is interesting to investigate whether or not a shorter intervention period is sufficient for patients to experience a positive impact on MIP and the degree of their dependency in activities of daily living, endurance in gait, fatigue, voice volume, phonation endurance and expiratory function*.* Especially, we find it valuable to investigate the effect of increasing intensity in rehabilitation as the intervention is relatively easy and inexpensive to implement and may be practiced by many patients on their own as an add-on to their rehabilitation.

A broad spectrum of patients with sensorimotor deficits following stroke is expected to be included in the study, as it does not exclude patients based on the severity of their stroke. We find it important to develop interventions that may also have an impact on the more severely injured patients.

Sham IMT was considered for the control group, but was rejected for ethical reasons, as it would be time consuming for patients to do the sham intervention, which would be unethical as these patients need to economise their resources due to fatigue.

An unpublished pre-study without a control group made in one part of the setting of this study indicated improvement in voice volume and phonation endurance not previously described as outcomes associated with IMT. Some patients in the pre-study described that these favourable outcomes enabled them to have phone conversations or longer face-to-face conversations with their relatives, giving them greater quality of life. We expect to obtain further knowledge on this previously undescribed field.

The pre-study has provided useful information about how to conduct the intervention, and the assessment points were adjusted according to what proved feasible in the pre-study.

## Trial status

Enrolment of participants starts on 01 February 2021. Recruitment, follow-up assessments and data analyses are expected to be completed by the end of December 2022. See Template (Additional file 1) and the Trials populated SPIRIT checklist (Additional file 2). Protocol version 2 (August 16, 2021)

## Supplementary Information

**Additional file 1.**

**Additional file 2.**

## Data Availability

During the data collection period for this study, the researchers and the assessors have access to the REDCap database. The assessors, however, have no access to information with regard to randomisation. The REDCap database automatically informs the assessors about trial progress completion. Only scientific investigators associated with this project will have access to data. The data collected in this study will be available from the principal investigator on reasonable request.

## Abbreviations

EF: Expiratory function
FFS: Fatigue Severity Scale
FIM: Functional Independence Measure
IMT: Inspiratory muscle training
mRS: Modified Rankin Scale
MIP: Maximal inspiratory pressure
RCT: Randomised controlled trial
VV&PE: Voice volume and phonation endurance
