# Supplementary material for: “More air—better performance—faster recovery”: study protocol for randomised controlled trial of the effect of post-stroke inspiratory muscle training for adults
Source: Trials. 2021 Aug 28;22:575. doi: 10.1186/s13063-021-05551-8 (PMC8401331; doi:10.1186/s13063-021-05551-8)
Supplement: Supplementary file 1 — Additional file 1. [file 13063_2021_5551_MOESM1_ESM.doc]

Additional file 1: Template of recommended contents for schedule of enrolment, interventions and assessments.

|  |  | | | | |
| --- | --- | --- | --- | --- | --- |
|  | **Enrolment** | **Allocation** |  | | |
| **TIMEPOINT** | ***-t_1_*** | **0** | ***t_1_*** | ***t_2_*** | ***t_3_*** |
| **ENROLMENT:** | 0-3 months after stroke |  |  |  |  |
| **Eligibility screen** | First week after hospitalisation at specialised neurorehabilitation unit |  |  |  |  |
| **Informed consent** | 2-4 days after hospitalisation at specialised neurorehabilitation unit |  |  |  |  |
| **Screening** | 4-6 days after hospitalisation at specialised neurorehabilitation unit |  |  |  |  |
| Randomisation | 4-6 days after hospitalisation at specialised neurorehabilitation unit | X |  |  |  |
| **INTERVENTIONS:** |  |  |  |  |  |
| **A: IMT** |  |  | x | x |  |
| **B: Conventional neurorehabilitation** |  |  | X | x |  |
| **ASSESSMENTS:** |  |  |  |  |  |
| ***Baseline:***  Inspiratory muscle training (MIP)  Functional independence Measure (FIM)  6-minute walking test  Fatigue Severity Scale (FSS)  Voice volume and phonation endurance (VV&PE)  Expiratory function (EF) |  |  | 0-4 days before the intervention |  |  |
| ***1: Follow-up:***  MIP, FIM, 6-minute walking test, FSS, VV&PE, EF and  Patient Global Impression of Change |  |  |  | Assessment 0-4 days after the intervention concluded |  |
| ***2. Second Follow-up***  MIP, FIM, 6-minute walking test, FSS, VV&PE and EF |  |  |  |  | Three months after the intervention concluded |
